# Translucency of recent zirconia materials and material-related variables affecting their translucency: a systematic review and meta-analysis

**DOI:** 10.1186/s12903-024-04070-7

**Published:** 2024-03-05

**Authors:** Mahinour Yousry, Ihab Hammad, Mohamed El Halawani, Moustafa Aboushelib

**Affiliations:** 1 Department of Conservative Dentistry, Faculty of Dentistry, Alexandria University, Alexandria, Egypt; 2 Department of Dental Materials Science, Faculty of Dentistry, Alexandria University, Alexandria, Egypt

**Keywords:** Cubic zirconia, Monolithic zirconia, Translucency, Optical properties, Highly translucent zirconia, Yttria stabilized zirconia

## Abstract

**Background:**

Recent forms of translucent zirconia material have been developed, offering a wide range of options and varieties for enhancing aesthetics, making it a preferred choice in the field of prosthetic dentistry. However, there is insufficient understanding regarding the recent types of zirconia materials and their optical behavior. Understanding the variables that influence the translucency of zirconia and identifying strategies to enhance its esthetics are crucial.

**Purpose:**

The current systemic review highlights a comprehensive understanding of different zirconia generations in relation to their optical characteristics and evaluates material-related variables affecting their translucency.

**Methods:**

The present review studied in-vitro studies that evaluated the optical characteristics of different yttria content of yttria stabilized materials. The topics explored were: (1) the different zirconia material generations and their optical behavior; (2) material-related factors that affect their translucency. The research was restricted to online publication in the English language from July 1, 2010, to July 31, 2023, using PubMed, Scopus, and Science Direct resources. The search key terms and their combinations were “zirconia,” “translucent zirconia,” “cubic zirconia,” “highly translucent zirconia,” “yttria partially stabilized zirconia,” “monolithic zirconia,” “translucency,” “optical properties,” and “light transmission.”

**Results:**

The data obtained from fifty-three studies addressed the optical characteristics of various zirconia generations. They reported that changing yttria content had a significant impact on translucency. Different kinds of zirconia ceramics of the same generation have varying translucencies. Achieving optimum aesthetics with monolithic zirconia is challenging due to factors related to material aspects such as the presence of additives, point defects, microstructure, thickness, phase distribution, and sintering conditions.

**Conclusions:**

Newly developed monolithic dental zirconia ceramics have improved aesthetics and translucency. However, additional research is necessary to evaluate their performance and long-term durability.

**Trial Registration:**

This systematic review was registered in PROSPERO, under number CRD42023474482.

**Supplementary Information:**

The online version contains supplementary material available at 10.1186/s12903-024-04070-7.

## Background

Achieving natural-looking dental restorations in dentistry can be challenging due to the complex optical characteristics of natural teeth. When light travels through a material, it undergoes partial reflection, scattering, and transmission. Translucency refers to the amount of light that can pass through a subject, and it is an important indicator of aesthetic outcomes of restoration. The degree of translucency is directly related to the amount of light that can pass through a material [1].

There are three numerical indicators used to assess the translucency of a material: the translucency parameter (TP), contrast ratio (CR), and light transmittance (T%). Johnston et al. were the first to use the translucency parameter as a direct way to measure translucency and analyze the optical properties of various dental materials [2, 3]. The TP is used to measure the color difference of a material against a black and white background, indicating its ability to mask different backgrounds. It is calculated using the equation [3, 4]:

$$TP = {[ (Lb* - Lw*) 2 + (ab* - aw*) 2 + (bb* - bw*) 2]}^{1/2}$$

L* refers to the degree of lightness between black (L = 0) and white (L = 100) and a range of gray shades in between. a* refers to redness and greenness [redness (a > 0) and greenness (a < 0)]. b* refers to yellowness to blueness [yellowness (b > 0) or blueness (b < 0) [3, 4]. A TP value of 100 indicates full transparency, while a value of 0 indicates complete opacity. The higher the TP value, the greater the translucency [5–8]:

$${\Delta E}_{00}={\left\{{\left[{\Delta L}^{\prime}/\left({k}_{L}{S}_{L}\right)\right]}^{2}+{\left[{\Delta C}^{\prime}/\left({k}_{C}{S}_{C}\right)\right]}^{2}+{\left[{\Delta H}^{\prime}/{k}_{H}{S}_{H}\right]}^{2}+{R}_{T}\left[{\Delta C}^{\prime}/\left({k}_{C}{S}_{C}\right)\right]\times \left[{\Delta H}^{\prime}/\left({k}_{C}{S}_{C}\right)\right]\right\}}^{1/2}$$

To account for differences in texture, background, and otherfactors, a new formula incorporates weighting functions (S_L_, S_C_, and S_H_) and parametric factors (k_L_, k_C_, and k_H_) for the lightness, chroma, and hue, respectively. According to Yu et al. (2009), [9] human dentin at 1.0 mm thickness has a TP value of 16.4, while enamel is 18.7. Restorative materials should closely mimic the aesthetic of natural teeth.

CR refers to the opacity of materials, and is defined as the ratio of luminous reflectance (Y) of a given material against a black (Yb) and white (Yw) backgrounds [2, 5, 10–12]. When the values of two luminous reflectance values are equal, a CR value of 0 referring to completely translucent and 1 referring to completely opaque. Materials are classified into four translucency classes based on their CR values [13]:

1. CR up to 0.50 is considered a highly translucent material.
2. CR 0.50 to 0.75 is considered a medium translucent material.
3. CR 0.75 to 0.90 is considered a low translucent material.
4. CR 0.90 to 1.00 is considered very low translucent materials (highly masking).

Another approach to assessing translucency is by calculating the transmission coefficient of light passing through a material, and This coefficient is determined by the ratio of specimen luminance to the source of luminance with wavelengths ranging between 400 and 700 nm. The measured spectrum of light transmittance ranges from 0% (completely opaque) to 100% (completely transparent) [14, 15].

Recent advancements have been made in enhancing the translucency of zirconia materials, particularly yttria partially stabilized zirconia (Y-PSZ) [16, 17]. Yttria partially stabilized zirconia (Y-PSZ) has considered now a highly demanded restorative material [18, 19]. The first generation of zirconia introduced in the late 1990s was the yttria-tetragonal zirconia polycrystal (3Y-TZP). It contains 0.25–0.5 wt% alumina, has superior mechanical qualities (a flexural strength of about 900–1200 MPa), but has insufficient translucency. It served as a framework and was covered with feldspathic porcelain [20]. The opaque color of 3Y-TZP and porcelain veneer chipping were the main drawback [21]. To overcome this problem, a second generation of monolithic (fully anatomic) 3% mol yttria partial stabilized zirconia (3YPSZ) was produced in 2011 [18, 22]. Enhancing the heat treatment conditions, reducing alumina content (0–0.2 wt% alumina), and increasing the sintering temperature, the cubic content increased from 6–12% to 20–30% and grain size increased to (0.5 –0.7 μm). As a result, translucency is enhanced while the biaxial strength is reduced from 1150 to 900 MPa [23]. Despite the improved translucency of this generation in comparison to the first one, It does not satisfy the higher aesthetic demands. Lithium disilicate exhibit greater translucency, yet it is considered to have lower mechanical characteristics compared to zirconia [24–26].

In 2014, a third generation of 5% mol yttria partial stabilized zirconia (5YPSZ) (high translucent) was introduced. This generation has an increase in yttria content (which contains up to 9.42 wt% in comparison to about 5.15 wt% for traditional zirconia) and an improvement in translucency due to the increased amount of the isotropic cubic phase. However, the flexural strength was reduced to 700–800 MPa [27]. In 2015, a multi-layered zirconia was created to accurately mimic the color gradient of natural teeth. Two varieties of multi-layered zirconia are available: (1) polychromatic multilayer uniform composition type (M5Y PSZ), where all layers gradually change in chroma from darker cervically to lighter incisal but have the same composition and opacity, and (2) polychromatic and hybrid composition multilayer (M3Y –5Y PSZ), where the layers vary in composition, chroma, and opacity [28].

In 2018, a fourth generation of PSZ (4Y PSZ), was developed, which falls between high-translucent zirconia (5Y PSZ) and high-strength (3Y PSZ) zirconia, having a flexural strength of 600 to 900 MPa. [18, 29] In addition, polychromatic multilayer type (M4Y) was introduced from 2018 to 2019. Recently, the fifth generation of ultra-high translucent zirconia (6Y-PSZ) and multilayered (M6Y-PSZ) were introduced, which have a higher yttria content (6 mol%), resulting in greater translucency but lower mechanical properties [18].

Overall, the evolution of zirconia materials in dentistry has provided practitioners with a wider range of options to meet the diverse needs of their patients. While previous researchers have conducted studies and provided classifications for dental zirconia, there is a lack of recent advances and updates to the optical behavior of these various types of highly translucent zirconia. Furthermore, the optical characteristics of zirconia could be affected by more than just its change in yttria concentration. Therefore, the current systematic review was conducted to provide a comprehensive and up-to-date overview of the recent types of translucent zirconia and to determine material-related factors that contribute to the translucency of zirconia material.

## Methods

**Fig. 1:**
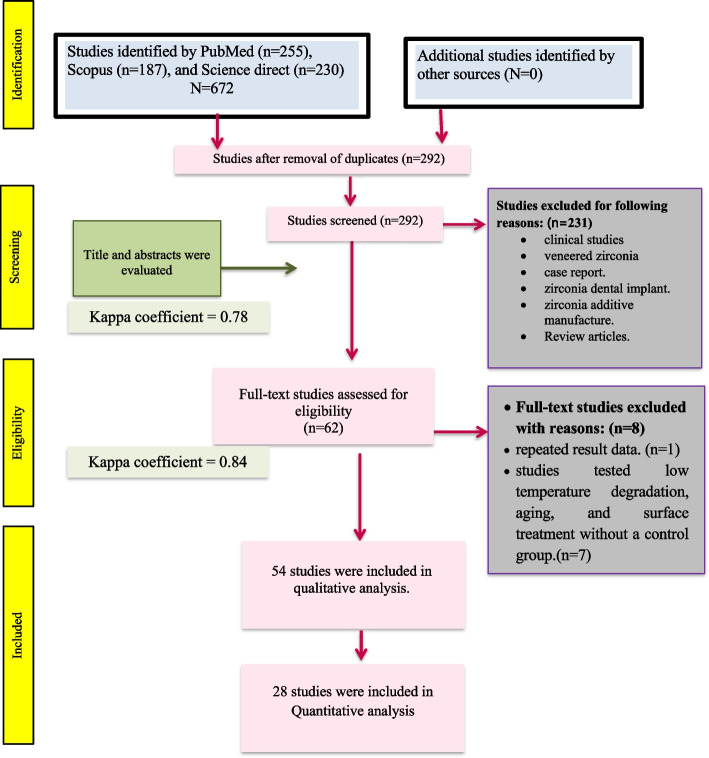
The search process and flow diagram following the Preferred Reporting Items for Systematic Reviews and Meta-Analyses (PRISMA) flowchart

This systematic review complied with the guidelines of the Preferred Reporting Items for Systematic Reviews (PRISMA) [30] and it was registered in the International Prospective Register of Systematic Reviews – PROSPERO under number CRD42023474482. Figure 1 summarizes the search strategy following the PRISMA guidelines.

The main PICO question [4] (P: population; I: intervention; C: control; O: outcome) was: (P) translucent zirconia with yttria content higher than 3 mol% yttria stabilized zirconia, (I) changing in yttria concentration in different zirconia materials would affect translucency. C: control group: 3% mol yttria stabilized zirconia. O: outcome of interest was the optical characteristics of these materials and the variables that influenced them.

### Eligibility criteria

All English in-vitro studies measuring translucency parameters, contrast ratio, or light transmission with uniform-thickness specimens were included in the review. As the sintering procedure is of such great significance for the densification behavior of zirconia, which reflects on the microstructure, mechanical, and optical properties of zirconia, the type of sintering used was mentioned in the review. Studies that combined zirconia core with veneering porcelain, zirconia veneer, crowns, implants, zirconia with a stabilizer other than Y2O3, or zirconia that has been processed via raw powder or additively manufactured zirconia were all excluded.

Studies that examine the optical characteristics of various layers within multilayer pre-colored zirconia, as well as external variables affecting the optical properties of zirconia material, including the underlying dental background, cement, or glazing, were not included in the study. In studies that examined how translucency changed after different processes, like aging, low-temperature degradation, or any kind of surface treatment, the current review only included the results of the control group that was not affected by the variables being tested.

### Search strategy

The search strategy used PubMed, Scopus, and Science Direct resources. The search was limited to English-language opened research articles published from 2010 to 2023. Boolean operators were used along with the following search keywords to identify relevant articles: “zirconia” OR “translucent zirconia” OR “cubic zirconia,” OR “highly translucent zirconia,” OR “yttria partially stabilized zirconia” OR “monolithic zirconia” AND “translucency” OR “optical properties” OR “light transmission”. The last database search was conducted on July 31, 2023. All identified keywords were adapted for each included information source and is provided in Appendix 1.

### Study screening and data extraction

Two reviewers (MY, MH) individually assessed the study titles and abstracts, choosing those that mostly satisfied the inclusion criteria for full study screening. The two reviewers conducted an independent evaluation of the chosen full-text publications, taking into consideration the predetermined inclusion and exclusion criteria. If there was disagreement, a third reviewer (MA) was consulted before the final decision on which papers to include had been made. Cohen’s kappa test was applied to assess the agreement between reviewers. The results were also expressed as the concordance between reviewers (%). The extracted data obtained for analysis were as follows: the authors, publication year, test method, material used/brand name, specimens tested number, sintering protocol used, specimen thickness, device used for optical measurement, and optical results. Extracted data were recorded using a Microsoft Excel spreadsheet (Microsoft Excel 2019 VL 16.44; Microsoft Corp., WA, USA).

#### Quality assessment

The risk of bias assessment of the included studies were performed using “Guidelines for Reporting Pre-Clinical In-Vitro Studies on Dental Materials” formulated by Faggion [31]. The risk of bias criteria were modified from prior studies [32–35], and assessed based on several parameters, including a structured summary in the abstract, the calculation of sample size, randomization of the specimens, an obvious description of sample preparation methods, adherence to the manufacturer’s recommendation for sintering, a clear description of methods used for optical properties, a clear reporting of outcomes with defined numbers and standard deviations, and statistical analysis used. The data-collection process involved using a predetermined table to evaluate the included articles. The ranking of each category ranged from 0 to 2, with 0; indicating clear reporting of the parameter by the author (s). 1; indicating that the author (s) reported the category but did not specify the parameter accuracy; and 2; if the author (s) have not obviously indicated the category or when the data is absent, the overall number of assigned values was documented, and every study was categorized based on the following criteria: studies with a sum between 0 and 4 were considered to have a low risk, those with a sum between 5 and 9 were considered to have a medium risk, and those with a sum between 10 and 14 were considered to have a high risk of bias. A predetermined table was used for data collection to evaluate the final articles.

### Meta-analysis

The systematic review did not include light transmittance since there was insufficient data for a meta-analysis. The outcome of translucency parameter and contrast ratio under different thicknesses (1.5, 1, 0.5, and 0.4 mm) were evaluated and compared between studies by meta-analysis using the RevMan software (Review Manager 5.4.1, The Nordic Cochrane Center, Copenhagen, Denmark) employing a random effect model [36]. Mean differences (MDs) and 95% confidence intervals (CIs) were used for the calculations. The control group was 3 mol yttria partially stabilized zirconia and the interventional groups contained more than 3 mol yttria (4,5, and 6 mol yttria). The p-values from the Q and I2 tests were used to identify heterogeneity; heterogeneity was determined to exist when the Q test’s *p*-value was less than 0.01. Heterogeneity was estimated to be minimal when the I2 test result was 25–50%; moderate when it was 50–75%; and high when it was greater than 75%. The findings were shown in the form of forest plots.

## Results

A schematic representation of the process employed for conducting the systematic review, complying with the guidelines outlined in the PRISMA statement, is represented in Fig. 1. The preliminary electronic searches conducted on PubMed, Scopus, and science direct resources yielded a total of 255, 187, and 230 studies, respectively. A total of 292 studies were primarily selected after the removal of duplicate studies to be assessed for their titles and abstracts. 231 studies were excluded as they did not fulfil the eligibility criteria. Consequently, only 62 studies remained and were chosen for a comprehensive analysis of their entire texts. One repeated result data and seven studies tested low temperature degradation, aging, and surface treatment without a control group; all were excluded. So, in the present systematic review, a total of 54 studies were incorporated. A 92% concordance with a Kappa coefficient of 0.78 (SE 0.082, 95% CI [0.619,0.941]) for titles and abstracts and a 93.5% concordance was found between the 2 reviewers, with a Kappa coefficient of 0.84 (SE 0.05, 95% CI [0.742, 0.938]) for full-text studies.

**Fig. 2:**
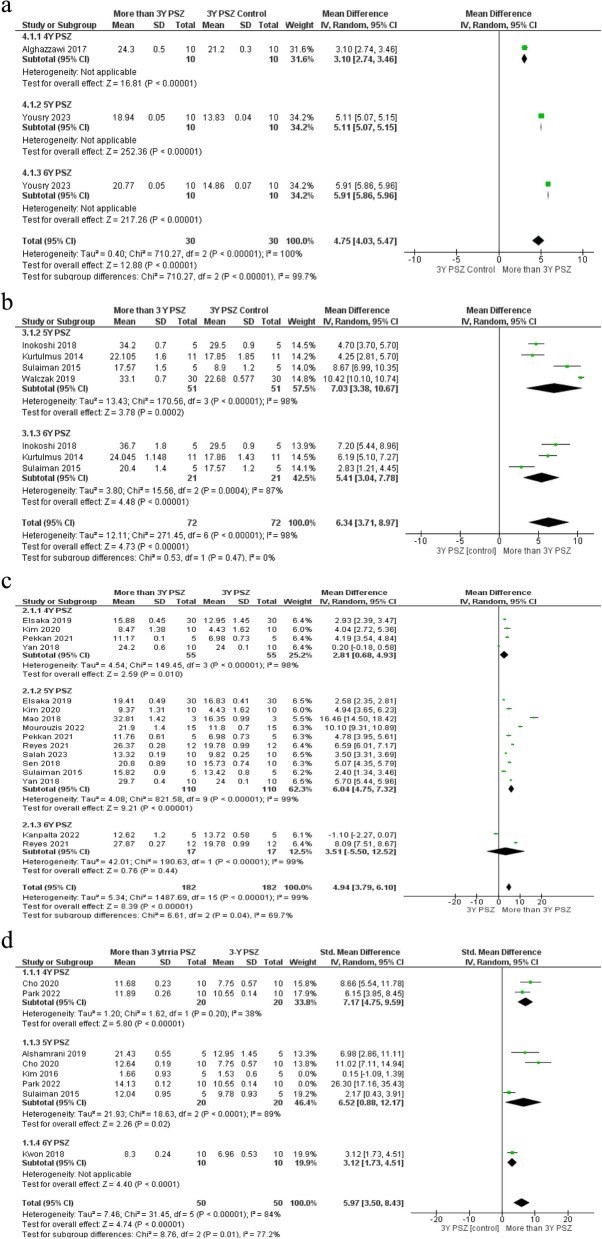
Forest plots based on the meta- analysis. **a** translucency parameter results with 0.4 mm zirconia thickness; **b** translucency parameter results with 0.5 mm thickness; **c** translucency parameter results with 1 mm zirconia thickness; **d** translucency parameter results with 1.5 mm zirconia thickness

**Fig. 3:**
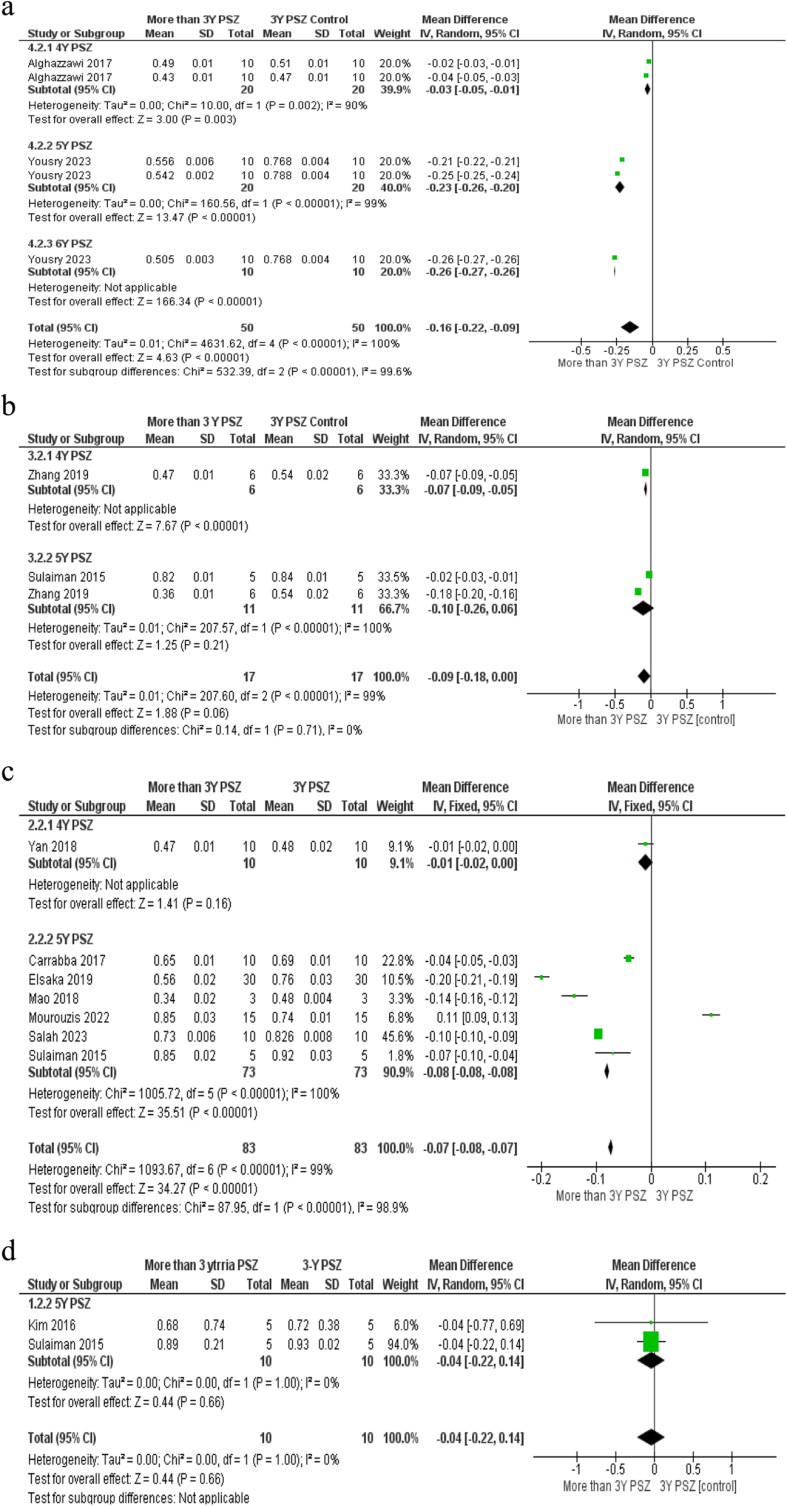
Forest plots based on the meta- analysis. Global **a** contrast ratio results with 0.4 mm thickness; **b** contrast ratio results with 0.5 mm thickness; **c** contrast ratio results with 1 mm thickness; **d** contrast ratio results with 1.5 mm thickness

A total of 35 studies exhibited a low risk of bias, while 17 studies revealed a medium risk of bias. Additionally, one article identified a high risk of bias. The assessment of the risk of bias for the included studies is demonstrated in Table 1. All the included studies provided sufficient data regarding the measurements of translucency, except for 10 studies that did not provide numerical values for optical measurement. A comprehensive detail of the studies included in the systematic review analysis is presented in Table 2, including information about each study’s authors, year, material brand names tested, kind of YPSZ, specimens tested number, type of sintering protocol used, specimen thickness, optical device used for measurement, and outcome results of the studies. In 22 studies, the number of tested specimens was 10, while 2 studies tested 12 specimens, 7 studies tested 15 specimens, 10 studies tested 5 specimens, and 3 studies tested 30 specimens. Only one study evaluated three specimens [37]. Luz et al. 2021 did not mention sample size [38]. Twenty-seven studies focused on evaluating different sintering protocols on the translucency. In 29 studies, the traditional CIE equation was used, while in 6 studies, the newest CIEDE2000 equation was applied. Twenty-two studies calculated the contrast ratio, and ten studies tested total transmittance. A full review of the classification of commercial zirconia materials and their brand names is presented in Table 3, as given by manufacturers or in connected references. The translucency parameter (TP) of 5 YPSZ for 1 mm thickness was varied from 9.37 to 29.7, TP of 3 YPSZ for 1 mm thickness was varied from 4.43 to 24, TP of 5 YPSZ for 0.5 mm thickness was varied from 20.4 to 36.7, and the CR of 5 YPSZ varied from 0.4 to 0.94. A total of 28 studies were incorporated into the meta-analysis. Studies that examined various kinds of YSZ or specimen thicknesses were counted on multiple occasions in the meta-analysis. The results showed high heterogeneity between studies except for the studies comparing 3 YPSZ and 5 YPSZ contrast ratio at thickness 1.5 mm. According to translucency parameter, there were statistically significant differences between control group and interventional groups (*P* value ≤ 0.0001) except between 3Y-PSZ and 4Y-PSZ at 1.5 mm thickness, to be 22.49 with a 95% confidence interval (CI) between 19.85 and 25.13. Meta-analysis found high heterogeneity of translucency parameter and contrast ratio between subgroups, I2 more than 75% (*p* < 0.0001) (Figs. 2 and 3).

**Table 1:** Assessment of risk of bias

Article	Structured summary in the abstract	Calculation of sample size	Obvious description of sample preparation methods	Randomization	Adherence to the manufacturer’s recommendation for sintering	Optical methods clearly described	Optical outcomes clearly reported	Total	Risk of bias
Salah et al. 2023 [39]	0	1	0	2	1	0	0	3	low
Yousry 2023 [40]	0	0	0	2	0	0	0	2	low
Savas and Akin 2022 [41]	0	0	0	2	1	0	0	3	low
Liu 2022 et al. [42]	0	0	0	2	1	0	2	5	medium
Liu 2022 et al. [43]	0	0	0	2	1	0	2	5	medium
Vafaei et al. 2022 [44]	0	1	0	2	1	0	0	4	low
Park et al. 2022 [45]	0	2	0	2	1	0	0	5	medium
Kongkiatkamon and peampring 2022 [46]	0	1	0	0	2	0	0	3	low
Mourouzis and Tolidis 2022 [47]	0	1	0	1	0	0	0	1	low
Kanpalta, Burduroglu and Kara 2022 [48]	0	0	0	0	0	0	0	0	low
Jerman et al. 2021 [49]	1	2	1	2	0	0	1	7	medium
Lümkemann and Stawarczyk 2021 [50]	1	2	0	2	0	1	1	7	medium
Reyes et al. 2021 [51]	0	2	0	2	0	0	0	4	low
Pekkan 2021 [52]	1	2	1	2	0	0	0	5	medium
Luz et al. 2021 [38]	1	2	1	2	1	1	2	10	high
Yang et al. 2020 [53]	1	2	1	2	0	0	2	8	medium
Cardoso et al. 2020 [54]	0	2	0	2	0	0	0	4	low
Cho et al. 2020 [55]	0	2	0	2	1	0	0	5	medium
Cokic et al. 2020 [56]	0	2	0	2	1	0	2	7	medium
Kim 2020 [57]	0	2	0	2	0	0	0	4	low
Sanal and Kilinc 2020 [58]	0	0	0	2	0	0	0	2	low
Lawson and Maharishi 2020 [59]	0	2	0	2	0	0	0	4	low
Aljanobi and Al-Sowygh 2020 [60]	1	2	1	2	0	0	2	8	medium
Zhang et al. 2019 [61]	1	1	0	2	0	0	0	4	low
Walczak et al. 2019 [62]	0	2	0	2	0	0	0	4	low
Alshamrani and Souza 2019 [63]	0	2	0	2	0	0	0	4	low
Elsaka 2019 [64]	0	2	0	2	0	0	0	4	low
Li et al.2019 [65]	0	2	1	2	1	0	2	8	medium
Jansen et al. 2019 [66]	0	2	1	2	0	0	2	7	medium
Juntavee and Attashu 2018 [67]	0	2	0	0	0	0	1	3	low
Yan et al. 2018 [68]	0	2	0	2	0	0	0	4	low
Liebermann et al. 2018 [69]	0	2	0	2	0	0	0	4	low
Inokoshi et al. 2018 [70]	0	2	0	2	0	0	0	4	low
Sen et al. 2018 [71]	0	2	0	2	0	0	0	4	low
Mao et al. 2018 [37]	0	2	0	2	0	0	0	4	low
Kwon et al. 2018 [72]	0	2	0	2	0	0	0	4	low
Zadeh et al. 2018 [73]	0	2	0	2	0	0	0	4	low
Alghazzawi 2017 [74]	0	2	0	2	0	0	0	4	low
Kim and Kim 2017 [75]	0	2	0	2	0	0	0	4	low
Carrabba et al. 2017 [76]	0	2	0	2	0	0	0	4	low
Stawarczyk et al. 2016 [77]	0	2	0	2	0	0	0	4	low
Kim and Kim 2016 [78]	0	2	0	2	0	0	0	4	low
Vichi et al. 2016 [79]	0	2	0	2	0	0	0	4	low
Abdelbary et al. 2016 [80]	0	2	0	2	0	0	0	4	low
Harada et al. 2016 [81]	0	2	0	2	0	0	0	4	low
Tuncel et al. 2016 [21]	0	2	0	2	0	0	0	4	low
Sulaiman et al.2015 [82]	0	2	0	2	0	0	0	4	low
Kurtulmus and Ulusoy 2014 [83]	0	2	0	2	0	0	0	4	low
Stawarczyk et al. 2014 [84]	0	2	0	2	0	0	0	4	low
Ebeid et al. 2014 [85]	0	2	2	2	0	0	0	6	medium
Kanchanavasita et al. 2014 [86]	1	2	1	2	0	0	0	5	medium
Stawarczyk et al. 2013 [87]	0	2	2	2	0	0	0	6	medium
Kim et al. 2013 [88]	0	2	2	2	0	0	0	6	medium
Jiang et al. 2011 [89]	0	2	2	2	0	0	0	6	medium

**Table 2:** A comprehensive overview of the optical properties observed in the included studies on different yttria-stabilized zirconia materials

Authors/ Publication Year	Test method	Material used/Brand name	Specimens tested number	Sintering protocol used	Specimen thickness	Device used for optical measurement	Optical results
Salah 2023 [39]	translucency parameter (TP) contrast ratio (CR) and with various sintering protocols	**5 YPSZ** (DD CubeX 2) **3 YPSZ** (DD Bio ZX2)	10	**Conventional sintering** 120 min holding time at 1450 C **Speed sintering** 50 min holding time at 1450 C **Superspeed** Sintering started at 1580 C with 10 min holding time	1 mm	spectrophotometer (Cary 5000 UV–Vis-NIR; Agilent Technologies)	**TP/CR** **DD CubeX 2**: Conv.13.32 ± 0.19/ 0.73 ± 0.006 Speed 11.5 ± 0.23/0.785 ± 0.004 Superspeed4.68 ± 0.2/0.913 ± 0.005 **DD Bio ZX2** Conv. 9.82 ± 0.25/ 0.826 ± 0.008 Speed 7.76 ± 0.4/0.879 ± 0.005 Superspeed 6.12 ± 0.22/ 0.898 ± 0.004
Yousry 2023 [40]	translucency parameter (TP) contrast ratio (CR) and with various sintering protocols	**3 YPSZ**	10	**Conventional and speed sintering** following the manufacturers’ instructions	0.4 mm	spectrophotometer (Vita Easy-Shade V; Vita Zahnfabrik)	TP00/CR InCoris: Conv. 14.86 ± 0.07/ 0.77 (0.004. Speed: 13.20 ± 0.08/0.79 ± 0.01 Lava: Conv: 13.83 ± 0.04/0.788 ± 0.01. Speed:11.07 ± 0.09/ 0.8 ± 0.005 Katana utml: Conv. 20.77 ± 0.05/ 0.505 ± 0.003 Speed: 19.52 ± 0.05/ 0.525 ± 0.005 Cercon Xtml: Conv. 18.94 ± 0.05/ 0.542 ± 0.002 Speed 17.58 ± 0.05/ 0.565 ± 0.005 Zolid:Conv. 17.03 ± 0.02/0.556 ± 0.006 Speed15.43 ± 0.27/ 0.574 ± 0.003
Savas 2022 [41]	translucency parameter TP_00_, contrast ratio CR after different sintering methods	**3YPSZ** InCoris TZI	10	**classic (C)**; (duration: 8 h), 1500 ^◦^C for 120 min **speed (S);** (duration: 130 min),1540 ◦C for 25 min **super-speed sintering (SS)** (duration: 130 min), 1580 ◦C for 10 min	1.5 mm	spectrophotometer (Vita Easy-Shade V; Vita Zahnfabrik)	TP_00_ Classic 7.37 ± 0.34 Speed 7.41 ± 0.48 Super-speed 7.10 ± 0.61 CR Classic 0.74 ± 0.015 Speed 0.73 ± 0.015 Super-speed 0.74 ± 0.022
Liu 2022 [42]	translucency parameter (TP) after different sintering protocols	**4Y-PSZ:** Katana HT, Zpex4, **5Y-PSZ:** Katana STML, Zpex Smile, **6Y-PSZ**: Katana UTML	15	conventional-sintering CS (1500 for 12 min, total time 7 h) speed-sintering SS (1515 C for30 mins, total time 90 min)	1.2 mm	colorimeter (CR-13; Konica-Minolta Sensing, Tokyo, Japan)	Results were shown in figures. Translucency (TP) was not significantly influenced by the sintering method - highest TP was Kat. UTML CS (25.40 ± 0.48)/ lowest TP Zpex Smile SS (15.34 ± 0.54)
Liu 2022 [43]	translucency TP of different sintering using two colorimeters	**3Y-PSZ**: Cercon HT (HT),Copran Zr-i Ultra-T (UT) **5Y-PSZ:** Cercon xt (XT),	5	Conventional and rapid sintering according to the manufacturers’ instructions	0.5, 0.8, and 1.2 mm	Vita Easy-shade V, zahinfabrik,Germany (Vita) Shadepilot,DeguDent, GmbH, Germany (DD)	Results were shown in figures TP values of the CS, RS group were nearly, except XT group with DD. XT was significantly highest TP
Vafaei 2022 [44]	translucency parameter (TP) with various sintering temperature	**5Y-PSZ**: -White Peaks Symphony (WPS) -e. max ZirCAD (IEZ)	10	at 1530, 1500, or 1440 °C with 17 °C/min heating rate for 2 h	1 mm	digital camera (Canon EOS 6D 20.2MP)	**(TP) WPS 1440:** 9.25 ± 0.99, 1500: 11.33 ± 0.68, at **1530:** 13.34 ± 0.83 **IEZ 1440:** 8.12 ± 0.47, 1500: 9.32 ± 0.25, at **1530:** 10.65 ± 0.48
Park 2022 [45]	translucency parameter (TP)	**5Y-PSZ**: Rainbow High Shine, **4Y-PSZ:**Rainbow Shine, **3Y-PSZ**:RainbowShade, lithium disilicate	10	sintered according to the manufacturers’ instructions	0.8 mm and 1.5 mm	spectrophotometer (SpectroShade Micro; MHT Optic Research AG)	**TP at 0.8 mm:** 3Y-TZP: 13.32 ± 0.32 4Y TZP: 14.12 ± 0.18 5Y TZP: 16.47 ± 0.39 Lithium disilicate 19.17 ± 0.29 **TP at 1.5 mm:**3Y-TZP: 10.55 ± 0.14 4Y TZP: 11.89 ± 0.26 5Y TZP: 14.13 ± 0.12 Lithium disilicate: 14.20 ± 0.32
Kongkiatkamon.2022 [46]	Effect of speed sintering on translucency parameter (TP)	5Y-PSZ: Katana STML	15	**RS: regular sintering** for 2 h, at 1550 ◦C **SS: speed sintering** for16 min, at1560 ◦C	1.23 mm	spectrophotometer (HunterLab, ColorQuest XE, Laboratory Inc., Reston)	**TP:** Regular Sintering10.581 ± 0.798 Speed Sintering 9.052 ± 0.618 **CR:** Regular Sintering = 0.787 ± 0.034 Speed Sintering = 0.833 ± 0.021
Mourouzis 2022 [47]	translucency parameter (TP_00_) and contrast ratios after three milling methods	**3Y-PSZ:** inCoris TZI **5Y-PSZ:** Katana STML	15	according to the manufacturer’s instruction Katana: dry-milled total sintering time18 min 1560 ºC, For the wet-milled: additional drying cycle at 200 ºC, for 12 min inCoris TZI: dry-milled total sintering time 28 min, 1580 ºC For the wet-milled: additional drying cycle at 200 ºC, for 3 min	1 mm	spectrophotometer Shimadzu UV (2401PC Series, UV–VIS)	**TP00**: **inCoris TZI: dry milling:** 11.8 ± 0.7 **Wet mill**:Distilled water:12.3 ± 0.3 **Impregnated water:** 10.7 ± 1.3 **Katana STML dry milling:** 21.9 ± 1.4 **Wet mill:**Distilled water 20.6 ± 0.4 **Impregnated water**:5.4 ± 1.2 **CR inCoris TZI dry milling:** 0.74 ± 0.1 **Wet mill**:Distilled water:0.92 ± 0.02 **Impregnated water** 0.94 ± 0.02 **Katana STML** dry milling: 0.85 ± 0.03 **Wet mill:**Distilled water: 0.90 ± 0.03 **Impregnated water:**0.96 ± 0.01
Kanpalta 2022 [48]	translucency parameter (TP)	lithium disilicate (IPS e.max CAD LT) **5YPSZ:**Prettau Anterior (PZA), VITA (YZ XT). **M6YPSZ** Katana UTML	5	various sintering temperatures 1550 C / 1450 C 2 h holding time	1 mm	spectrophotometer VITA Easyshade Advance 4.0; VITA Zahnfabrik)	**TP: UTML:** At 1450; 12.11 ± 0.41 At 1550 12.62 ± 1.2 **PZA**: At 1450; 14.01 ± 0.64 At 1550; 14.88 ± 1.91 **YZ XT:** At 1450; 13.73 ± 0.19 At; 1550; 13.72 ± 0.58 **IPS:** 18.93 ± 0.52
Jerman 2021 [49]	Light transmittance	Zirconia (pritidenta GmbH): O opaque (**3YPSZ**), T translucent (**3YPSZ**),ET extra translucent (**4YPSZ**), HT high translucent (**5YPSZ**)	15	1450 ◦C with 2 h holding time	1.45 mm ± 0.04 mm	UV/Vis spectrophotometer (LAMBDA 35, PerkinElmer LAS, Germany)	Light transmission coefficient percentage: O = 26.6/ T = 31.6 ET = 33.7 / HT = 35.3
Lümkemann 2021 [50]	Light transmittance	**Y-PSZ**0.25 (Ceramillzi), **3YPSZ**0.05 (CeramillZ), **5YPSZ** (Ceramill Z fx), **Pre 4YPSZ** (Ceramill Z ht + Preshades) **4YPSZ** (Ceramill Z ht +)	30	according to the manufacturers’ instructions conventionally sintered 5Y-TZP, 3Y-TZP 0.05, 3Y-TZP 0.25, and 4Y-TZP were at 1450 C high-speed sintered for pre4Y-TZP, 4Y-TZP at 1580 C	1.2 mm	an UV/Vis spectrophotometer (LAMBDA 35, PerkinElmer LAS, Germany)	Light transmittance% 5Y-TZP = 23.7 ± 0.6 3Y-TZP 0.25 = 9.5 ± 0.4 3Y-TZP 0.05 = 12.1 ± 0.4 pre 4Y-TZP speed = 13.1 ± 1.3 4Y-TZP speed = 0.1 ± 0.0 4Y-TZP = 16.8 ± 0.4 LiSi 2 = 34.0 ± 0.9
Reyes et al., 2021 [51]	translucency parameter (TP)	lithium disilicate (IPS e.max, Press HT, LT) **3YPSZ:** BruxZir, **5YPSZ:** BruxZir Anterior, Katana STML **M6YPSZ** Kat UTML	12	followed the manufacturers’ instructions	1 ± 0.05 mm	A spectrophotometer (CM-2600D; Minolta,Inc)	**TP: BruxZir:** 19.78 ± 0.99, **BruxZir Anterior:** 25.33 ± 0.75**, Kat UTML**: 27.87 ± 0.27**, Kat STML**26.37 ± 0.28 **e.max LT** 32.85 ± 0.84, **e.max HT**: 37.34 ± 1.4
Pekkan 2021 [52]	translucency parameter (TP) with various sintering protocols and thicknesses	**3Y-PSZ** CopranZri (CZI) **4Y-PSZ** CopraSupreme (CSP), **5Y-PSZ** CopraSmile (CSM)	5	120 min at 1500 °C, slow programme (SLP) 90 min at 1500 °C on the normal rate (NRP) 30 min at 1500 °C.Speed program (SPP) 120 min at 1600 °C Translucency program (TRP)	0.7, 1.0, 1.3 mm	a chromometer (Minolta CM- 2300d series; Minolta Sensing, Inc.)	TP: **CZI:** **Slow program (SLP):** 0.7 mm 8.64 ± 0.45 1 mm 7.50 ± 0.78 1.3 mm 6.84 ± 1.29 **Normal program (NRP):** 0.7 mm 8.63 ± 0.29 1 mm 6.98 ± 0.73 1.3 mm 5.26 ± 0.37 **Speed Program (SPP)** 0.7 mm 8.22 ± 0.29 1 mm 6.41 ± 0.13 1.3 mm 5.09 ± 0.13 **Translucency program (TRP):** 0.7 mm 9.96 ± 0.25 1 mm 7.90 ± 0.16 1.3 mm 6.66 ± 0.16 **CSP:** Slow program (SLP): 0.7 mm 12.62 ± 0.16 1 mm 11.13 ± 0.14 1.3 mm 9.91 ± 0.12 Normal program (NRP): 0.7 mm 12.75 ± 0.15 1 mm 11.17 ± 0.10 1.3 mm 9.64 ± 0.36 Speed Program (SPP) 0.7 mm 13.07 ± 0.21 1 mm 1.24 ± 0.13 1.3 mm 9.73 ± 0.14 Translucency program (TRP 0.7 mm 11.73 ± 0.47 1 mm 11.45 ± 1.65 1.3 mm 10.79 ± 0.83 **CSM:**Slow program (SLP): 0.7 mm 13.78 ± 0.20 1 mm 12.00 ± 0.35 1.3 mm 10.62 ± 0.11 Normal program (NRP): 0.7 mm 13.02 ± 0.42 1 mm 11.76 ± 0.61 1.3 mm 10.82 ± 0.49 Speed Program (SPP) 0.7 mm 11.59 ± 0.49 1 mm 10.43 ± 0.78 1.3 mm 9.40 ± 0.40 Translucency program (TRP 0.7 mm 13.37 ± 0.25 1 mm 11.86 ± 0.46 1.3 mm 10.92 ± 0.48
Luz 2021 [38]	Translucency parameter (TP)	**3Y-TZP** Vipi Block	Not mentioned	**Conventional sintering:** 1530 ◦ C / 10 h **microwave speed sintering:**1450 ◦ C /1 h 45 min	1.2 mm	Not mentioned	TP: **Conventional sintering**: 29 ± 0.8 **microwave speed sintering:** 13 ± 1
Yang 2020 [53]	Translucency parameter (TP)	**3Y-TZP**: Copran Zr **3Y-PSZ**:Copran Zr-I Ultra-T, Copran Zr-I Ultra-T white, CecronHT **5Y-PSZ**: Cecron XT	5	**Conventional sintering CS:** 130 min/1520 ◦C:Cecron HT, XT, 90 min/1500 ◦C: Copran Z, Zr-I Ultra-T, Zr-I Ultra-T white **Speed sintering SS:** 35 min/1540 ◦C: Cecron HT, XT 90 min/ 1500 ◦C: Copran Z, Zr-I Ultra-T, Zr-I Ultra-T white	1-mm	A spectrophotometer (Cary 5000 UV–Vis-NIR; Agilent Technologies)	The result did not mention in numerical values. RS process has a different impact on the optical qualities -significant differences between CS &SS
Cardoso 2020 [54]	translucency parameter (TP)	**5Y-PSZ** Prettau Anterior	15	2 h dwell time, at 1450 C and at 1600 C	1.2 ± 0.01 mm	spectrophotometer (CM 2600d; Konica Minolta Sensing Inc)	**TP:** At 1450, 15.45 ± 0.18 At 1600 15.58 ± 0.37
Cho2020 [55]	Translucency parameter (TP)	lithium disilicate (Rosetta SM) **4Y- PSZ** (Katana STML), **3Y- PSZ** (Katana HT) **5Y-PSZ** (Katana UTML)	10	according to the manufacturer’s recommendations	0.8 and 1.5 mm	a spectrophotometer (SpectroShade Micro)	**TP: at 0.8 mm: HT** 11.58 ± 0.57**, STML**13.90 ± 0.17**/ UTML** 15.36 ± 0.5 **Rosetta SM** 19.18 ± 0.29 **At 1.5 mm** **HT** 7.75 ± 0.57**, STML** 11.68 ± 0.23 **UTML**12.64 ± 0.19 **Rosetta SM**14.20 ± 0.39
Cokic 2020 [56]	Contrast ratio CR / translucency parameter TP	**5Y-PSZ**:Katana STML, **3Y-PSZ**:inCoris TZI, CEREC Zirconia	7	STMLSS: 1560 ◦C /30 min and (CEREC ZrSS)1578 ◦C/15 min STML CS 1550 ◦C/6.8 h and inCoris TZICS 1510 ◦C/4 h	3.5 mm	a spectrophotometer (SpectroShadeTM MICRO, MHT Optic Research)	Results in figures higher TP for Kat STMLSS and Kat STMLCS than CEREC ZrSS and inCoris TZICS -CEREC ZrSS TP = 15
Kim2020 [57]	Light transmittance (T%), translucency parameter (TP)	**3Y-TZP** (Luxen Zr, **4Y-PSZ** (Luxen Enamel), **5Y-PSZ** (Luxen Smile), IPS e.max CAD	10	S: air-sintered at 1500C for two hours before being air-cooled rapidly cooled RS: at 1500C for one hour then rapidly air-cooled within 1–2 min	1 mm	a spectrophotometer (Ci7600; X-Rite, Grand Rapids)	**3Y-TZP:** TP: S = 4.43 ± 1.62, T%30.86 (5.82) RS = TP: 4.65 ± 0.39, T%:32.59 (4.73) **4Y-PSZ:** S = TP:8.47 (1.38), T%44.13 (5.27) RS = TP: 8.93 ± 0.43, T%:49.39 (2.99) **5Y-PSZ:** S = TP: 9.37 ± 1.31, T%:51.08 (4.38) RS = TP:9.66 ± 1.06, T%:53.94 (1.06) **e.max CAD**: TP: 17.42 ± 0.29, T%: 86.17 (0.91)
Sanal2020 [58]	translucency parameters (TP00) with different temperatures,shades and thicknesses	**5Y-PSZ** Katana STML	10	Three sintering temperatures (1350 C, 1450 C, and1600 C) with 2 h dwell time	(1 mm-1.5 mm	A spectrophotometer (Vita EasyShade, Vita Zahnfabrik)	**TP00 shade a2** **1 mm:** at 1350:7.55 ± 0.86, at 1450:8.31 ± 0.27, at 1600:8.6 ± 0.77 **1.5 mm:** at 1350, 4.98 ± 0.36, at 1450: 6.93 ± 0.35, at 1600:6.77 ± 1.37 **shade a3** **1 mm**: at 1350: 7.2 ± 0.8, at1450: 8.35 ± 0.22, at 1600: 9.11 ± 0.22 **1.5 mm**: at 1350:4.99 ± 0.64, at 1450:6.56 ± 0.47, at 1600:7.42 ± 0.52
Lawson2020 [59]	translucency parameter (TP00) with different sintering	**5YPSZ:** KatanaSTML, Prettau Anterior and Zpex Smile IPS e.max CAD	10	conventional 7-h preprogrammed speed sintering 30 min preprogrammed speed sintering 18 min	1 mm	spectrophotometer (Color-i7; X-Rite, Grand Rapids)	TP_00_ Katana STML (18 min) 7.64 ± 0.20 Katana STML (30 min) 7.61 ± 0.25 Katana STML (7 h) 7.88 ± 0.25 Prettau Anterior (30 min 3.96 ± 0.26 Prettau Anterior (7 h) 7.88 ± 0.27 Zpex Smile (30 min) 5.17 ± 0.12 Zpex Smile (7 h) 8.47 ± 0.17 IPS e.max CAD 9.33 ± 56
Aljanobi [60] 2020	Translucency parameter (TP)	**5YPSZ:** Prettau, 2Dispersive, PrettauAnt -IPS e.max CAD HT	12	manufacturer’s instructions	1 mm	a spectrophotometer (LabScan XE, Hunter Associates Lab. Inc.)	TP results in figures -Different TP among materials Emax significantly highest TP
Zhang2019 [61]	Contrast ratio (CR)	**3Y-PSZ** Zpex **4Y-PSZ** Zpex4 **5Y-PSZ** ZpexSmile	6	1450 °C holding time 2 h	0.5 mm	spectrophotometer (SpectroShadeTMMICRO,OpticResearch)	Zpex 0.54 ± 0.02 Zpex4 0.47 ± 0.01 ZpexSmile 0.36 ± 0.01
Walczak 2019 [62]	contrast ratio (CR), (TP) translucency parameter	**3YPSZ:** BruxZir Solid **3YPSZ:** Zenostar **3YPSZ:** Lava Plus	30	manufacturer’s instructions	0.50 ± 0.01 mm	A spectrophotometer (Gretag SPM 100; Gretag Limited)	**BruxZirTP**:11.66 ± 0.73 **CR:**0.76 ± 0.01/ **Zenostar:TP**12.96 ± 0.89, **CR**0.74 ± 0.18 **Lava Plus: TP**10.59 ± 0.72,**CR**0.79 ± 0.14
Alshamrani/ 2019 [63]	translucency parameter (TP)	**5YPSZ:** Ceramill Zolid FX / **3YPSZ:** e.max Zircad	5	1200 C, holding time 2 h	1.5 mm	a spectrophotometer (CR-321;Minolta Co. Ltd.)	**TP** Ceramill Zolid FX: 21.43 ± 0.55 IPS e.max Zircad: 12.95 ± 1.45
Elsaka 2019 [64]	contrast ratio (CR), translucency parameter (TP)	5YPSZ Prettau Anterior (PA), Ceramill Zolid FX Multilayer (CZF), 4YPSZ:ZenostarT (ZT)	30	manufacturer’s recommendations	1 mm	spectrophotometer (CM-2006d;Konica Minolta)	**CZF:** TP 19.41 ± 0.49,CR:0.56 ± 0.02 **PA**: TP 16.83 ± 0.41,CR: 0.74 ± 0.03 **ZT**: TP 15.88 ± 0.45,CR: 0.76 ± 0.03
Li 2019 [65]	transmittance with different sintering protocols	3Y-PSZ: ST Preshaded UPCERA Zirconia	10	**Total time:** **CS:** 15 h **CS‐R:**40 min, **R‐1:** 12 min, **R‐2:** 25 min **R‐3: 40 min**	0.5 mm	a spectrophotometer (Color i7800, Xrite)	Results were shown in figures lowest light transmittance is R1 -transmittance is similar between CS and R‐2 and R‐3
Jansen et al. 2019 [66]	Light transmittance	3Y- PSZ: Zolid (ZD) and Ceramill ZI 4Y- PSZ: Zolid HT +	10	2 Speed sintering (1570 C and 1590 C) Conventional sintering (1450 C)	1.5, 2.0, 2.5, and 3.0 mm	a UV–Vis spectrophotometer (LAMBDA 35)	results were shown in figures sintering protocols had no significant effect on the translucency of ZI Translucency significantly decreased for ZD and HT + with speed sintering
Juntavee 2018 [67]	Contrast ratio (CR), translucency parameter (TP)	3Y-PSZ: VITA YZ HT	15	various sintering (HP, 180 min), (HR, 120 min), (HS,60 min) and various temperatures: (SD, 1350 °C), (SI, 1550 °C), (SR, 1450 °C)	1.5 mm	spectrophotometer (ColorQuest XE, Hunter Associated Laboratory)	TP, CR Mean ± sd (95% confidential interval CI) for SDHS: TP 1.4 ± 0.13, CR 0.982 ± 0.004, for SDHR TP:2.16 ± 0.10, CR: 0.967 ± 0.005 for SDHP TP:2.24 ± 0.10, CR:0.964 ± 0.004 for SRHS: TP: 3.03 ± 0.10, CR:0.945 ± 0.003 for SRHR TP 3.19 ± 0.17, CR: 0.942 ± 0.003 for SRHP TP: 3.42 ± 0.10, CR:0.937 ± 0.003 for SIHS: TP:3.16 ± 0.09, CR: 0.937 ± 0.002 for SIHR TP:3.05 ± 0.20, CR: 0.939 ± 0.005 for SIHP.TP:2.95 ± 0.18, CR: 0.942 ± 0.006
Yan/ 2018 [68]	translucency parameter (TP), Contrast ratio (CR)	5Y-PSZ: Zpex Smile 4Y-PSZ: Zpex 4 3Y- PSZ: Zpex IPS e.max CAD	10	Zpex: 1530 °C Zpex 4:1450 °C Zpex Smile: 1450 °C	1.0 ± 0.2 mm	SpectroShade Micro; MHT	Zpex TP: 24.0 ± 0.1,CR: 0.48 ± 0.00 Zpex 4 TP:24.2 ± 0.6, CR: 0.47 ± 0.01 Zpex Smile TP: 29.7 ± 0.4 0.37 ± 0.00 IPS e.max CAD: TP = 34.3 (0.9) CR = 0.37 (0.01)
Liebermann 2018 [69]	light transmittance	3 Y-TZP Bruxzir (BX), Lava Frame (LF), Cercon (CE), **3Y-PSZ:** Zenostar (ZS), Prettau (PT), Lava Plus (LP) Lithium disilicate:LS2 (EM)	20	recommended by the manufacturer	LF,LP 0.3,1 mm; PT &BX 0.5,1 mm; ZS & CE 0.4,1 mm; BX 0.5,1 mm EM1mm	A spectrophotometer (CM-2006d, Germany)	Light transmittance EM 1 mm 44.72 ± 0.005 LF 0.3 mm 40.19 ± 0.004 PT 0.5 mm 33.54 ± 0.005 BX 0.5 mm 39.59 ± 0.008 CE 0.4 mm 38.52 ± 0.006 ZS 0.4 mm 33.95 ± 0.005 LP 0.3 mm 41.15 ± 0.006 BX 1 mm 31.61 ± 0.008 LF 1 mm 26.26 ± 0.002 LP 1 mm 28.09 ± 0.003 PT 1 mm 25.94 ± 0.014 ZS 1 mm 19.64 ± 0.003 CE 1 mm 25.30 ± 0.007
Inokoshi, 2018 [70]	translucency parameter (TP)	**3YPSZ:** Katana HT **5YPSZ:** KatanaSTML, **5YPSZ:** Zpex Smile. **6MYPSZ:**KatanaUTML	5	1550 C with 2 h Dwelling time	0.5 mm	a colorimeter (CR13; Konica-Minolta Sensing)	**TP:** Kat UTML (36.7 ± 1.8), Kat HT (29.5 ± 0.9), Zpex Smile (33.1 ± 0.7), Kat STML (34.2 ± 0.7)
Sen 2018 [71]	translucency parameter (TP)	**3YPSZ:** Vita YZHTColor (VYZb), VitaYZHT White (VYZa), Prettau Zirkonzahn (PZ) **5Y- PSZ:** Prettau Anterior (PZA)	10	Final sintering temperatures (1350 C, 1450 C, and 1600 C)	1.0 ± 0.05 mm	spectrophotometer, (Color Eye 7000A Xrite; GretagMacbeth)	**VYZa colored:**1350 C 15.28 ± 0.43, 1450 C 17.14 ± 0.71, 1600 C 18.26 ± 0.36 **VYZb precolored**1350 C 17.28 ± 0.56, 1450 C 18.03 ± 0.87, 1600 C 18.40 ± 0.27 **VYZa noncolored:**1350 C 16.42 ± 0.62, 1450 C 17.49 ± 0.38, 1600 C 18.05 ± 0.44 **PZ colored:**1350 C 14.37 ± 0.27 1450 C 15.73 ± 0.74 1600 C 16.74 ± 0.46 **PZ noncolored**1350 C 14.86 ± 0.21 1450 C 16.05 ± 0.36 1600 C 16.32 ± 0.28 **PZA colored:**1350 C 18.96 ± 0.65 1450 C 21.34 ± 1.04 1600 C 22.76 ± 0.62 **PZA noncolored:**1350 C 19.23 ± 0.4 1450 C 20.80 ± 0.89 1600 C 22.03 ± 1.55
Mao/ 2018 [37]	contrast ratio, translucency parameter TP	5Y- PSZ: Zpex Smile 3Y- PSZ: Zpex	3	followed the manufacturer’s instruction	1 mm	Colorimeter (SpectroShade Micro; MHT)	**ZpexTP**16.35 ± 0.99, **CR**0.48 ± 0.004 **Zpex Smile TP**32.81 ± 1.42, CR0.34 ± 0.02
Kwon 2018 [72]	translucency parameter TP_00_	**3YPSZ:** Katana HT **M6-YPSZ:**Katana utml e.max CAD LT, HT	10	followed the manufacturer’s instruction	1.5 mm (e.max 1.1 mm)	a spectrophotometer (CM-700d; Konica)	**TP**_**00**_** Kat utml** (8.30 ± 0.24), **Kat HT** (6.96 ± 0.53), **e.max CAD HT** (12.64 ± 0.48) **e.max CAD LT** (9.28 ± 0.36)
Zadeh 2018 [73]	Light transmittance (Tt%)	**5Y- PSZ:** DD cubeX2, CopraSmile, Ceramill Zolid FX, NOVAZIR MaxT, StarCeram Z‑Smile Priti multidisc ZrO 2, IPS e.max Press	10	followed the manufacturer’s instruction	1 mm	spectrophotometer (Lambda 35; PerkinElmer LAS)	Light transmittance (Tt%): Ceramill Zolid FX 38.3 ± 0.3 CopraSmile 37.1 ± 0.3 NOVAZIR MaxT 33.1 ± 0.5 DD cubeX2 37.3 ± 0.3 StarCeram Z‑Smile 33.6 ± 0.2 priti multidisc ZrO 2 37.6 ± 0.5 IPS e.max Press 40.4 ± 0.4
Alghazzawi et al. 2017 [74]	Contrast ratio (CR), Translucency parameter (TP)	**3Y- PSZ:** Zenostar ZR, ZirluxFG2, Bruxzir Solid **4Y- PSZ:** Katana HT, Nexx ZrT	10	followed the manufacturer’s instruction	0.4 mm	Spectrophotometer (Crystaleye, ModelCE 100-DC/US, v1.3.1.0; Olympus Corp)	**ZirluxFG2 TP**: 21.2 ± 0.3, **CR**:0.47 ± 0.01, **KatanaHT TP**: 24.3 ± 0.5, **CR**: 0.43 ± 0.01, **NexxZrT TP**:20.1 ± 0.7, **CR**: 0.49 ± 0.01, **Zenostar ZR: TP**24.1 ± 0.4,**CR**:0.41 ± 0.01 **Bruxzir Solid TP**: 21.2 ± 0.4, **CR**:0.50 ± 0.01
Kim and Kim 2017 [75]	Translucency parameter TP_00_ with different sintering	3Y- PSZ: Rainbow Shade	9	Conventional: 8 hs /1500 ◦ C holding 2 h Microwave: 2 hs/1500 °C holding 30 minuted	0.5, 1.0, 1.5 mm	a spectrophotometer (Color iControl, X-Rite)	**TP**_**00**_ results shown in figures statistically significant differences among thicknesses and sintering
Carrabba 2017 [76]	contrast ratio (CR)	5Y- PSZ:Aadva NT[NT] 3Y- PSZ:Aadva EI [EI] 3 Y-TZP:Aadva ST [ST], IPS e.max CAD LT[LD]	10	followed the manufacturer’s instructions	1.0 ± 0.1 mm	a spectrophotometer (PSD1000, OceanOptics)	**CR:** LD 0.56 ± 0.02 ST 0.74 ± 0.01 NT0.65 ± 0.01 EI 0.69 ± 0.01
Stawarczyk/ 2016 [77]	contrast ratio (CR)	3 Y-TZP:Ceramill ZI **3Y PSZ:** Zenostar, DD BioZX2, Ceramill Zolid, InCoris TZI	15	followed the manufacturer’s instructions	0.5	A spectrophotometer (CM-2600d, Konica)	**CR: Zenostar:** 0.57 0.01**, DD Bio ZX 2:** 0.62 ± 0.01**, Ceramill Zolid:** 0.57 ± 0.01**, InCoris TZI:** 0.57 ± 0.01**, Ceramill ZI:** 0.77 ± 0.01
Kim et al. 2016 [78]	contrast ratio (CR)	3Y PSZ: Rainbow Shade (A05, A2), Upcera‑ST A1,A2,A3 5Y PSZ: Rainbow High Shine A0, A1,A2 e.max CADLT,HT (A1,A2,A3)	5	followed the manufacturer’s instructions	1.5 mm	spectrophotometer (Color i5, X-Rite)	Rainbow Shade: A05 = 1.53 ± 0.6/ A2 = 0.61 ± 0.11 ‑Rainbow High Shine: A0 = 1.66 ± 0.93, A1 = 1.68 ± ,0.74,A2 = 2.31 ± 0.35 Upcera‑ST: A1 = 0.79 ± 0.43,,A2 = 0.72 ± 0.38, A3 = 0.56 ± 0.15 e.max LTA1 13.75 ± 1.87/ e.max LTA2 15.63 ± 0.20/ e.max LTA3 12.83 ± 2.26/ e.max HTA1 19.78 ± 2.88/ e.max HTA2 22.41 ± 0.21/e.max HTA3 22.66 ± 0.30
Vichi 2016 [79]	contrast ratio (CR), translucency parameter (TP),	3 Y-TZP: inCoris ZI, VITA In-Ceram YZ) **3YPSZ:** IPS e.max Zir-CAD, inCorisTZI, VITA In-Ceram YZ HT	10	according to manufacturer instruction	1.2 mm	spectrophotometer (PSD1000, OceanOptics)	**e.max ZirCAD**TP:11.48 ± 0.53,CR 0.75 ± 0.01. **inCorisZI:**TP12.64 ± 0.93**/** CR:0.74 ± 0.02**, inCorisTZI**: TP14.05 ± 0.31 / CR:0.68 ± 0.01 **In-Ceram YZ** TP:13.78 ± 0.28**/** CR: 0.70 ± 0.01**. In-CeramYZ HT:**TP: 14.44 ± 0.34**/** CR: 0.68 ± 0.01
Abdelbary 2016 [80]	translucency parameter TP	3Y- PSZ: inCoris TZI	15	90 min and 1540° C sintering temperature	0.5, 0.8, 1, 1.2 mm	spectrophotometer (Vita EasyShade)	TP 0.5 mm = 16.12 0.8 mm = 13.67, 1 mm = 11.49 1.2 mm = 9.25
Harada 2016 [81]	Light transmittance	3YPSZ:BruxZir, 4YPSZ:Katana HT 5YPSZ:Prettau Anterior, M5Y- PSZ:Katana ST M6Y-PSZ:Katana UT e.max CAD LT	5	followed the manufacturer’s instructions	0.5, 1.0 mm	spectrophotometer (Evolution 300 UV–Vis; ThermoFisher	**Light transmittanceT% 0.5/1.0 mm** **BruxZir** 28.82 ± 0.22/ 20.13 ± 0.22 **Kat HT** 28.49 ± 0.14/ 20.18 ± 0.39 **Prettau Ant.** 31.88 ± 0.49/ 22.58 ± 0.41 **Kat UT** 33.73 ± 0.13/ 23.37 ± 0.27 **Kat ST** 31.67 ± 0.24/ 21.86 ± 0.14 **E-max LT** 40.32 ± 0.25/ 27.05 ± 0.56
Tuncel 2016 [21]	contrast Ratio (CR)	3Y- PSZ: Prettau Zirconia	5	1600 °C final temperature, with 2 h holding time	0.5 mm	spectrophotometer (Vita Easyshade Compact,Bad Sackingen)	**CR** 0.796 (± 0.004)
Sulaiman et al.2015 [82]	Translucency parameter (TP) contrast Ratio (CR)	3 Y-TZP: ICE Zircon (ICE) 3YPSZ: Prettau (PRT), Zenostar (ZEN), Bruxzir (BRX), Katana (KAT), fully stabilized zirconia (5Y-FSZ): Prettau Anterior	5	followed the manufacturer’s instructions	0.5, 0.7, 1.0, 1.2, 1.5, and 2.0 mm	reflection spectrophotometer (CM-700d, Konica Minolta Sensing Inc)	**TP values/ CR values** **ICE: 0.5 = **16.59 / 0.86, **0.7**mm14.41 / 0.88, **1.0**mm11.47/ 0.90, **1.2** mm 9.92/ 0.92, **1.5**mm8.52/ 0.93, **2.0** mm 6.38/ 0.95 **PRT: 0.5**mm17.13/ 0.85**, 0.7** mm 15.5/ 0.87**, 1.0mm**12.46/ 0.90, **1.2 mm** 10.62/ 0.92**, 1**.5mm8.73/ 0.93, **2.0 mm** 6.38/ 0.95 **PRTA 0.5mm**20.4/ 0.82**, 0.7mm**17.6/ 0.84**, 1.0mm**15.82/ 0.85**, 1.2mm**14.82/ 0.86**, 1.5mm**12.04/0.89 **2.0mm**9.74/ 0.91 **BRX: 0.5mm**17.76/ 0.86**, 0.7mm**15.03/ 0.89, **1.0mm**12.32/ 0.92, **1.2mm**10.53/ 0.93**, 1.5mm**8/ 0.95, **2.0mm**5.65/ 0.97 **ZEN: 0.5mm**8.9/ 0.84**, 0.7mm**15.91/ 0.86**, 1.0mm**13.95/ 0.88**, 1.2mm**11.84/ 0.90**, 1.5mm**9.47/ 0.92 **2.0mm**7.46/ 0.98 **KAT: 0.5mm**17.57/ 0.84, **0.7mm**15.11/ 0.85**, 1.0mm**13.42/ 0.87**, 1.2mm**11.69/ 0.89**, 1.5mm**9.78/ 0.91**, 2.0mm**7.78/ 0.93
Kurtulmus-Yilmaz S 2014 [83]	Translucency parameter (TP)	in A3.5, A2,A1shades **3Y-PSZ:**Katana (KTN), In-Ceram YZ (VYZ), ICE Zirkon (ICE), IPS e.max Press (IPS)	11	followed the manufacturers’ instructions	0.5 mm	ITA Easyshade Compact spectrophotometer	**TP VYZ: A1**: 22.68 ± 0.577 **A2** 21.98 ± 1.076, **A3.5** 22.54 ± 0.815 **ICE: A1:** 17.86 ± 1.430 **A2:** 17.85 ± 1.851,**A3.5** 17.065 ± 1.761 **KTN:A1** 24.045 ± 1.148 **A2** 22.105 ± 1.606, **A3.5** 19.065 ± 1.289
Stawarczyk 2014 [84]	contrast Ratio (CR)	3 Y-TZP:Lava (LZ),ICE Zirkon (IZ),Vita In Ceram YZ (VI), InCoris ZI (IC), CopranYZ (CY), DD BioZ (DD),ZENO Bridge (ZE),Cercon (CC), **3YPSZ:**CeramillZi (CZ), GC DiscCIP (GC), Prettau (PR) / glass ceramic:VitaMarkII (CG)	12	according to manufacturers’ instruction	0.5 ± 0.05 mm	spectrophotometer (CM-2600d, Konica Minolta)	**CR:** CC (0.85 ± 0.01).CG: (0.58 ± 0.01); PR (0.74 ± 0.01), VI (0.76 ± 0.01), LZ (0.74 ± 0.01), GC (0.75 ± 0.01), IC (0.81 ± 0.01), CY (0.78 ± 0.01), DD (0.78 ± 0.02), CZ (0.77 ± 0.01), IZ (0.76 ± 0.03)
Ebeid 2014 [85]	contrast Ratio (CR)	3Y- PSZ: Bruxzir,	10	holding time (4, 2, 1 h) sintering temperature (1600 ◦C, 1530 ◦C,1460 ◦C)	1 mm	spectrophotometer (Easyshade compact, Vita Zahnfabrik)	Contrast Ratio (CR) At 1460, holding time 1 h 0.75 ± 0.02 At 1460, holding time 2 h 0.75 ± 0.03 At 1460, holding time 4 h 0.71 ± 0.01 At 1530, holding time 1 h 0.72 ± 0.01 At 1530, holding time 2 h 0.71 ± 0.01 At 1530, holding time4h 0.69 ± 0.01 At 1600, holding time 1 h 0.71 ± 0.01 At 1600, holding time 2 h 0.70 ± 0.01 At 1600, holding time4h0.68 ± 0.01
Kanchanavasita 2014 [86]	contrast Ratio (CR)	3Y- PSZ:Cercon Base,	10	according to the manufacturers’ recommendations	0.3, 0.6, 0.9, 1.2, 1.5 mm	A spectrocolorimeter (ColorFlex, Model 45/0; Hunter Lab, Inc.)	CR:Cercon Base: 0.3 mm 0.76 0.6 mm 0.84, 0.9 mm 0.91 1.2 mm 0.97, 1.5 mm 0.99
Stawarczyk et al. 2013 [87]	Ratio (CR) After different sintering temperatures contrast	3Y- PSZ: Ceramill ZI	10	120 min holding time, various final temperatures: 1700 °C, 1650 °C, 1600 °C, 1550 °C, 1500 °C,1450 °C, 1400 °C, 1350 °C, or 1300 °C	0.7 mm	a spectrophotometer (CM-2600 d, Konica Minolta)	CR 1,300 °C, 0.85 ± 0.01 1,350 °C, 0.81 ± 0.01 1,400 °C, 0.78 ± 0.01 1,450 °C, 0.77 ± 0.01 1,500 °C, 0.77 ± 0.02 1,550 °C, 0.75 ± 0.01 1,600 °C, 0.74 (0.01 1,650 °C, 0.70 ± 0.01 1,700 °C, 0.68 ± 0.01
Kim et al.2013 [88]	light transmittance	3 Y-TZP:Lava Frame, 3Y- PSZ: KaVo Everest ZS	10	Microwave sintering (MS) 20 min dwell time and conventional sintering (CS) 2, 10, or 40 h dwell time	1 mm	spectrophotometer. (SpectraMagic CM-3500d, Konica Minolta)	light transmittance (%) **Lava:** CS – 20 min 30.32 ± 0.64 CS – 2 h 29.80 ± 0.32 CS – 10 h 28.86 ± 0.16 CS – 40 h 28.39 ± 0.19 MS – 20 min 34.48 ± 0.24 **KaVo:** CS – 20 min 29.62 ± 0.20 CS – 2 h 28.61 ± 0.31 CS – 10 h 28.39 ± 0.43 CS – 40 h 28.09 ± 0.37 MS – 20 min 30.50 ± 0.37
Jiang 2011 [89]	light transmittance	3 Y- PSZ: TZ-3YB-E	10	Various sintering temperatures 1500, 1450, 1400, and 1350 °C	0.50 ± 0.01 mm	spectrometer with a double-prism monochromator (Bentham Instr. Ltd.)	As the temperature went up from 1,350 to 1,500 °C, light transmittances increased too

**Table 3:** Shows the different types of zirconia materials

**Zirconia generation**	**Brand Name**	**Manufacturer**
**1) Uniform composition and monochromic**		
First generation Y−TZP	inCoris Zi	Dentsply Sirona
	Z-CAD HD	Metoxit AG
	dima Mill Zirconia ST	Kulzer GmbH
	Aadva ST	GC Corp
	Copran Zri	Whitepeaks Dental Solutions GmbH & Co. KG
	Lava Frame	3 M ESPE, St. Paul, MN
	DD Bio Z	Dental Direkt GmbH
	ceramill zi	Amann Girrbach AG
	Vita YZ T	Vita Zahnfabrik H. Rauter GmbH & Co. KG
	Cercon base	Dentsply Sirona
Second generation 3YPSZ	Vita YZ HT	Vita Zahnfabrik H. Rauter GmbH & Co. KG
	IPS e.max ZirCAD MO/LT	Ivoclar Vivadent AG
	inCoris TZI	Dentsply Sirona
	Aadva EI	GC Corp
	Prettau	Zirkonzahn GmbH
	Cercon ht	Dentsply Sirona
	ceramill zolid	Amann Girrbach AG
	Z-CAD HTL	Metoxit AG
	Lava Plus	3 M ESPE, St. Paul, MN
	Zpex	Tosoh Corporation
	Rainbow	Genoss, Suwon
	Luxen Zr	Dentalmax, Seoul, Korea
	BruxZir	Glidewell Direct
	DD Bio ZX2	Dental Direkt GmbH
	Zenostar	Wieland Dental Technik, GmbH & Co.KG
	UPCERA ST zirconia	Shenzhen Upcera Co
	KaVo Everest ZS	KaVo Dental GmbH
	ICE Zirkon Translucent	Zirkonzahn, Italy
	Zeno Z	Wieland Dental Technik, GmbH & Co.KG
Third generation 5YPSZ	Aadva NT	GC Corp
	CopraSmile	Whitepeaks Dental Solutions GmbH & Co. KG
	Priti multidisc ZrO 2	Pritidenta,GmbH
	Prettau Anterior	Zirkonzahn GmbH
	Zpex Smile	Tosoh Corporation
	Rainbow High Shine	Genoss, Suwon
	Luxen Smile	DENTALMAX, Seoul
	BruxZir Anterior Solid Zirconia	Glidewell Direct
	NOVAZIR MaxT	Novadent Dentaltechnik
	Z-CAD Smile	Metoxit AG
	Ceramill zolid fx	Amann Girrbach AG
	StarCeram Z-Smile	H.C. Starck, Masan High-Tech Materials Group
	Lava Esthetic	3 M ESPE, St. Paul, MN
	DD cube X2	Dental Direkt GmbH
	Vita YZ XT	Vita Zahnfabrik H. Rauter GmbH & Co. KG
	Cercon xt	Dentsply Sirona
Fourth generation 4YPSZ	DDcube ONE	Dental Direkt GmbH
	Katana Zirconia HT	Kuraray Noritake Dental Inc
	Z-CAD One4All	Metoxit AG
	Rainbow Shine T	Genoss, Suwon
	Zpex 4	Tosoh Corporation
	Zenostar T	Wieland Dental Technik, GmbH & Co.KG
	Vita YZ ST	Vita Zahnfabrik H. Rauter GmbH & Co. KG
	ceramill zolid HT +	Amann Girrbach AG
	IPS e.max ZirCAD MT	Ivoclar Vivadent AG
	CopraSupreme	Whitepeaks Dental Solutions GmbH & Co. KG
	Luxen Enamel	DENTALMAX
6YPSZ	Katana Zirconia UT	Kuraray Noritake Dental Inc
2) Uniform composition and polychromic multilayer (M)		
M3YPSZ	Dima Mill Zirconia ML	Zirkonzahn GmbH
	Prettau 2 Dispersive	Kulzer GmbH
	Nacera Pearl Multi-Shade	Doceram Medical Ceramics GmbH
M4YPSZ	Katana Zirconia ML	Kuraray Noritake Dental Inc
	DDcube ONE ML	Dental Direkt GmbH
	Ceramill zolid gen-x	Amann Girrbach AG
	Z-CAD One4All Multi	Metoxit AG
	Vita YZ ST Multicolor	Vita Zahnfabrik H. Rauter GmbH & Co. KG
	Shofu Block Zr Lucent CEREC CopraSupreme Symphony	Shofu Inc./Adamant Namiki
		Whitepeaks Dental Solutions GmbH & Co. KG
M5YPSZ	Z-CAD Smile Multi	Metoxit AG
	DD cube X^2^ ML	Dental Direkt GmbH
	Ceramill zolid fx multilayer	Amann Girrbach AG
	CopraSmile Symphony	Whitepeaks Dental Solutions GmbH & Co. KG
	Vita YZ XT Multicolor	Vita Zahnfabrik H. Rauter GmbH & Co. KG
	Katana Zirconia Block STML	Kuraray Noritake Dental Inc
	Prettau 4 Anterior Dispersive	Zirkonzahn GmbH
	Cercon xt ML	Dentsply Sirona
	Lucent FA	Shofu Inc./Adamant Namiki
M6YPSZ	Nacera Pearl Q^3^ Multi-Shade	Doceram Medical Ceramics GmbH
	Katana Zirconia UTML	Kuraray Noritake Dental Inc
3) Hybrid composition and polychromic multilayer (M)		
M3Y-5YPSZ	Prettau 3 Dispersive	Zirkonzahn GmbH
	IPS e.max ZirCAD Prime	Ivoclar Vivadent AG
	Tanaka Enamel ZR Multi 5	ATD Japan Co., Ltd
	Lucent Supra	Shofu Inc./Adamant Namiki
	Zivino	Yoshida Dental Co., Ltd./Adamant Namiki
M4Y-5YPSZ	CopraSupreme Hyperion	Whitepeaks Dental Solutions GmbH & Co. KG
	IPS e.max ZirCAD MT Multi	Ivoclar Vivadent AG
M3-4Y PSZ	Sakura Zirconia	Straumann Japan/Adamant Namiki

## Discussion

The current review presents several studies that have been conducted to examine the optical characteristics of zirconia. However, the findings of these studies have shown high heterogeneity in results seen in some analyses, which can be attributed to a combination of factors, including variances in the instruments used, the wide range of materials tested, the differences in methods used for sample preparation, and the large number of covariables that are associated with heterogeneity. Most of these studies focused on the translucency parameter test. This may be due to the direct calculations in the translucency parameter method, while light transmittance and CR methods have been utilized in spectral or luminous conditions [90]. Furthermore, the translucency parameter values of dentin and enamel can be used as a reference when comparing the values reported in the literature. As it was estimated by Yu 2009 [9], the translucency parameter value of 1 mm of dentin was 16.4 and that of enamel was 18.7. Park 2022, Cho 2020, found that the translucency parameter of highly translucent zirconia (5Y PSZ) at a thickness of 0.8 mm was nearly that of lithium disilicate and dentin [45, 55]. Mourouzis 2022 [47], Reyes 2023 [51], Elsaka 2019 [64], Sen 2018 [71] and Mao 2018 [37] found that the translucency parameter of highly translucent zirconia (5Y PSZ) had a value range from 32.81 to 18.95 at a thickness of 1 mm, which was higher than the estimated TP value of human dentin or enamel at 1 mm.

The control of desired color and aesthetic qualities in zirconia dental restorations is a complex process that can be influenced by a combination of material and clinical variables [13, 79, 82, 83, 86–88, 91, 92]. The clinical variables include the underlying tooth structure, cement layer, surface structure, thickness, light sources, glazing, external staining, and the influence of different surface treatment conditions [93]. The current systematic review focused on the material-related variables that affect the translucency of yttria-stabilized zirconia materials, which were the influence of sintering additives, point defects, thickness, sintering condition, microstructure, density, t'-phase, coloring effect, manufacturing processes (blank fabrication), and coloring effect.

### Influence of sintering additives

#### Amount of yttria content (3% or 5–8%)

A common method is adding yttria content to the zirconia composition at a higher percentage to stabilize it, which results in an increased cubic phase and improved translucency. This correlation is related to the isotropic (uniform) feature of the cubic zirconia structure, which allows light to pass through more easily [23, 55, 70, 76]. In comparison to 3Y, the translucency of 5Y is enhanced by 20 to 25% [81].

#### Alumina content

A change in the aluminum percentage had a notable impact on translucency. When the aluminum exceeded 0.41%, the translucency decreased, as demonstrated by Walczak et al. 2019 [62], Elsaka et al. 2019 [64], Sen et al. 2018 [71], Carrabba 2017 [76], and Vichi et al. 2016 [79] as it has a different refractive index than zirconia (*n* = 1.76 for alumina and *n* = 2.21 for zirconia [94]. Reducing alumina content to below 0.05% has no noticeable effect on the material’s mechanical properties [81]. The total removal of alumina content is unnecessary, as the alumina-free 3Y-TZP had similar translucency to the 3Y-0.05 alumina content ceramic [23].

### Point defects

In the Y-TZP lattice, defects like oxygen vacancies can form, which were considered intrinsic features of oxides. These lead to an increased absorption coefficient of light and decreased light transmission (increased opacity). Oxygen vacancies can influence the color of zirconia. Based on the concentration and arrangement of oxygen vacancies, zirconia can exhibit different colors [95]. Controlling and manipulating oxygen vacancies in zirconia is an active area of research, as it allows tailoring the material’s properties for specific applications. Techniques like doping with specific elements, optimizing processing conditions, or using nanoscale engineering approaches can be employed to modulate the concentration and behavior of oxygen vacancies in zirconia [95, 96]. It is recommended to do after sintering air-based heat treatment (annealing) at 750 °C to get back oxygen into the crystal structure to get rid of the oxygen vacancies, which lowers the absorption coefficient and allows more transmission of light (increases translucency). However, there would be no change in the grain size or the true porosity at this low temperature [97].

### Sintering condition

Several studies focused on evaluating the optical properties of zirconia after different sintering conditions, as it played a significant role in determining optical and mechanical properties [39, 41–44, 46–48, 50, 52–54, 56–59, 65–68, 71, 75, 85, 87–89].

Sintering regulations such as final sintering temperature, total sintering duration, heating rate, and dwell time determine the microstructure, density, grain size, material stability, porosity, and crystalline content of zirconia [67, 84, 87, 88, 98].

#### Sintering temperature

Stawarczyk et al. 2013,2014 [84, 87] stated that an elevated final sintering temperature results in enhanced translucency through increased grain size, reduced porosity, a more compact crystalline structure, and increased density of zirconia. An increase in the grain size of zirconia was observed when the sintering temperature exceeded 1,300 °C. However, grain expansion caused neighbouring grains to be compressed, and hollow voids formed in the zirconia microstructure when sintering temperatures exceeded 1,600 °C.

#### Sintering time

There is debate regarding how different sintering times affect the translucency of zirconia. Several studies suggested speed sintering procedures resulted in a reduction in translucency. that further investigations are necessary to find out the effect of different sintering methods on various YSZ [42, 43, 52, 53, 56, 65, 66, 75]. Salah 2023 found that superspeed sintering for 10 min significantly reduced translucency and resulted in a greater change in color [39]. The findings of a study conducted by Lawson 2020 [59] indicated that speed sintering led to an increase in average grain size and pore formation, which decreased the translucency. On the other hand, conventional sintering took longer time, resulting in growth, segregation of the grains, and decreased porosity. Contradictory results were found in previous investigations by Kim 2013 and Vichi 2016, that reducing the sintering process decreases the grain size, which raises the light transmittance of Y-TZP [79, 88]. Microwave sintering was found to improve properties such as larger grain size and greater color value, whereas conventional sintering enhanced translucency slightly [75].

### Microstructure

#### Grain size

Zhang et al. 2012 [99] stated that the preferred grain size is 80 nm or less to increase the translucency of a zirconia ceramic to mimic that of dental porcelains. On the contrary, Jiang et al. 2011 observed that increasing the grain size from 40 to 90 nm, decreased the translucency of zirconia [89]. and this was in agreement with Kim 2013 and Vichi 2016 [79, 88]. Zhang suggested that for optimal translucency, a thickness of 2 mm would require a grain size of 70 nm, and a thickness of 1.3 mm would require a grain size of 82 nm [94]. Another study claimed that translucency is dependent on the average grain size and the number of grain boundaries. Reduced grain size leads to an increase in grain boundaries, which in turn causes a decrease in translucency. The increased sintering temperature of Y-TZP causes an increase in grain size and enhanced translucency [54, 58, 67, 71, 85, 87–89, 100].

#### Pores and grain boundaries

Pores are primarily responsible for the occurrence of light scattering, especially if their size closely matches those of visible light wavelengths, which range between 400 and 700 nm [65, 101]. Interaction with light is caused by different refractive indices at various interfaces, including grain/pore, grain/grain, and distinct crystallographic anisotropic grains. Porosities contribute to light scattering and reduce translucency since air has a refractive index of *n* = 1 and zirconia has a refractive index of *n* = 2.1–2.2 [102]. Translucency can be reduced if the size of the porosity is between 200 and 400 nm and the porosity contents are as low as 0.05%. Through the manipulation of sintering parameters, including an increase in temperature and time, porosities can be reduced [84, 87].

### Phase distribution

The tetragonal zirconia crystal possesses an anisotropic crystalline structure, which leads to birefringence or optical anisotropy (a single incoming ray is refracted in two directions). This means that due to the dissimilar crystal orientation of adjacent grains in the zirconia structure, the refractive index (∆n) breaks at the boundaries, resulting in the scattering of light [102]. This light scattering led to both refraction and reflection at grain boundaries, with alterations in the incident light beam direction and a resultant diminishment in the light transmittance [54, 58, 71, 85, 87, 89, 94].

### t’-phase

By changing the phase composition through a specific cooling process, it was possible to make a translucent monolithic zirconia that contained t’-phase without adding any dopant elements. Kim et al. 2020 found that a rapid-cooling protocol enhanced the translucency of 3–5 mol% Y-PSZ. They attributed that to the formation of t’-ZrO2, which contributes to improving the light that passes through the zirconia, making it more transparent [57]. The material attained a stable state through annealing at 1550 ◦C, and during the rapid cooling process, the c-phase changed into the t’-phase without the t-phase changing into the m-phase. The c-phase underwent a diffusionless transition to the t’-phase, which remained stable at room temperature. The t’-phase in translucent zirconia is formed through a diffusionless mechanism where oxygen ions in the lattice parameter are displaced. Due to this displacement, new domains are created that have crystal parameters close to those of the isotropic structure. This characteristic improves translucency by minimizing birefringence-induced light scattering [57].

### Density

To achieve 95% of its theoretical density, the sintering temperature of yttria-stabilized zirconia (YSZ) needs to be raised to 1350 °C. However, a sintering temperature of 1500 °C ensures that zirconia will attain its theoretical density [87, 89, 103]. Furthermore, it has been observed that the density of the material increases when the heating rate is raised from 50 °C to 100 °C. However, it should be noted that above 100 ◦C, the density decreases because of the rapid aggregation of the particles, leading to inadequate densification and pore formation [79, 104].

### Thickness

A negative correlation was observed between material thickness and translucency [66]. For highly translucent zirconia, the optimal mechanical and aesthetic range of thickness is likely to be between 0.5 and 1 mm, while a clinical range of 0.5 to 0.75 mm may be considered acceptable for conventional zirconia monolithic restorations [70].

The translucency of e-max CAD LT was higher by 20% than that of 5-YPSZ and 6-YPSZ specimens at 0.5- and 1-mm thickness; however, the 1 mm e-max CAD LT specimen exhibited a lower level of translucency than 0.5 mm of 5-YPSZ and 6-YPSZ. For successful monolithic restorations with reduced occlusal thickness and minimal tooth reduction, 5-YPSZ, 6-YPSZ could be utilized as alternatives to lithium disilicate that requires a thicker occlusal layer for optimal outcomes [81]. Recent studies demonstrated that 5 mol% yttria-partial stabilized zirconia and lithium disilicate exhibited similar levels of translucency [81, 105] which made high-translucent zirconia to be used with a reduced thickness than lithium disilicate in high-aesthetic areas [28, 72, 81, 105]. Increased TP values were found for conventional sintering at 1 and 0.5 mm, while the sintering procedure had no effect on the TP values at 1.5 mm [75].

### Manufacturing processes (Blank fabrication)

For manufacturing monolithic ZrO_2_ blanks, the ZrO_2_ powder is first grounded to decrease the particle size and then combined with a binder to get rid of the closed pores, increase the density, and compact the green body. This method improves the monolithic ZrO_2_ light transmission and allows for higher natural shade, as stated by Vichi et al. (2016) who stated that the chemical purity of the powder, granule properties, pressing sort, and treatment before sintering are all important aspects of this manufacturing procedure that determine the final characteristics [79].

### Coloring effect

Use of pre-colored blocks, immersion of white zirconia in coloring solutions, or painting of the restorations are all viable options for coloring monolithic zirconia restorations. Elsaka 2019 compared the optical characteristics of multilayer and monochromatic monolithic zirconia. Multilayered zirconia exhibited significantly increased TP and decreased CR values in comparison to monochromatic zirconia; these differences were correlated with the greater number of grains present in the multilayer zirconia [64]. Carrabba 2017, compared three uncolored of different Y-TZP, there were statistically significant differences between groups [76]. Sen et al. 2018 found that the translucency of the noncolored and precolored groups was significantly higher than the translucency of colored YPZ sintered at a final temperature of 1350◦C. Coloring liquid had no effect on the translucency of YPZ, whereas it decreased the translucency of fully stabilized translucent zirconia [71]. Kim 2016 [78] concluded that TP values varied significantly between various shades of the same zirconia brand. There were statistically significant variations in TP values between brands, even for a corresponding shade.

Based on the findings of this review, clinicians are advised to know the recent variations of zirconia and understand the differences among its various types. Furthermore, the management of the desired translucency and color characteristics of zirconia restoration is a complex procedure that can be affected by an interaction of internal factors related to the material used, as well as external factors associated with the substrate and surroundings of the restorations. Understanding these factors aids in choosing the appropriate type of zirconia according to the clinical condition.

According to the present literature, monolithic translucent zirconia has shown promising esthetic outcomes, making it a suitable choice for cases requiring esthetics. However, some limitations were present since detailed data could not be fully obtained on the external variable affecting the translucency of zirconia. Also, the review compared different in vitro studies that used various types of zirconia. However, further research on clinical trials with extended follow-up periods should be conducted to acquire stronger evidence and support the findings.

## Conclusions

Within the limitations of this study, it was concluded that recent research has explored the application of yttria partly stabilized zirconia with varying yttria percentages to enhance its translucency. However, achieving the desired translucency and color characteristics of zirconia restorations is a complex process not only influenced by yttria percentage. It can also be affected by interactions with other internal factors related to the material used, as well as external factors associated with the substrate and surroundings of the restorations.

### Supplementary Information

**Supplementary Materials 1. **

## Data Availability

All data generated or analyzed from this study are included in this published article.
