# Supplementary material for: Translucency of recent zirconia materials and material-related variables affecting their translucency: a systematic review and meta-analysis
Source: BMC Oral Health. 2024 Mar 5;24:309. doi: 10.1186/s12903-024-04070-7 (PMC10913643; doi:10.1186/s12903-024-04070-7)
Supplement: Supplementary file 1 — Supplementary Materials 1. [file 12903_2024_4070_MOESM1_ESM.docx]

**Supplemental Table 1.** Electronic search strategy.

| **Data source** | **terms and/or keywords** | **limit** |
| --- | --- | --- |
| **PubMed** | **#1** " zirconia "[MeSH Terms]  **#2** " zirconia "[ Title/Abstract] OR " translucent zirconia"[Title/Abstract] OR " cubic zirconia "[Title/Abstract] OR " highly translucent zirconia "[Title/Abstract] OR " yttria partially stabilized zirconia "[Title/Abstract] OR " monolithic zirconia "[Title/Abstract].  **#3** " translucency "[Title/Abstract] OR " optical properties "[Title/Abstract] OR " light transmission "[Title/Abstract].  **#4** “Dental Implant” [MeSH Terms] OR “Crown”[MeSH Terms]  **#5** **#1 AND #2 AND #3 NOT #4** | **English / research article** |
| **Scopus** | **#1:** TITLE-ABS-KEY (("zirconia" OR " translucent zirconia" OR " cubic zirconia" OR " highly translucent zirconia" OR " yttria partially stabilized zirconia " OR " monolithic zirconia "))  **#2:** TITLE-ABS-KEY (("translucency" OR " optical properties " OR " light transmission))  #**3:** TITLE-ABS-KEY ((zirconi* OR "Yttria-stabilized zirconia"))  #**4: #1 AND #2 AND #3** | **English / research article** |
| **Science Direct resources** | (“zirconia” OR “translucent zirconia” OR “cubic zirconia,” OR “highly translucent zirconia,” OR “yttria partially stabilized zirconia” OR “monolithic zirconia”) AND (“translucency” OR “optical properties” OR “light transmission”). | **English / research article** |

.
